# Effects of vitamin C and E on toxic action of alcohol on partial hepatectomy-induced liver regeneration in rats

**DOI:** 10.3164/jcbn.17-96

**Published:** 2018-04-03

**Authors:** Yurika Okamura, Akira Omori, Norihiko Asada, Akifumi Ono

**Affiliations:** 1 Faculty of Medical Nutrition, Hiroshima International University, 5-1-1 Hirokoshingai, Kure city, Hiroshima 737-0112, Japan; 2 Department of Food and Nutrition, Toyama College, 444 Gankaiji Minakuchi, Toyama 930-0193, Japan; 3 Department of Food and Nutrition, Beppu University, 82 Kita-Ishigaki, Beppu, Oita 874-8501, Japan; 4 Department of Clinical Nutrition, Faculty of Health Science and Technology, Kawasaki University of Medical Welfare, 288 Matsushima, Kurashiki-City, Okayama 701-0193, Japan

**Keywords:** partial hepatectomy, liver regeneration, alcohol, oxidative stress, antioxidant

## Abstract

The purpose of this study was to investigate the influence of vitamins C and E on the toxic action of alcohol in rat liver regeneration. Male Sprague-Dawley rats subjected to 70% partial hepatectomy were divided into five groups (Groups 1 to 5). Rats in Groups 2 to 5 were only provided alcohol for drinking. Additionally, vitamin C, vitamin E, and vitamin C in combination with vitamin E were administered to Groups 3, 4, and 5, respectively. Alcohol inhibits liver regeneration, resulting in an increase in free radicals produced by alcohol metabolism and thus causing cellular damage and altering liver function. During liver regeneration, vitamins C and E significantly ameliorated liver injury from alcohol administration by reducing hepatic lipid peroxidation. Vitamins C and E protect against liver injury and dysfunction, attenuate lipid peroxidation, and thus may be more effective in combination than either vitamin alone against alcohol-mediated toxic effects during liver regeneration.

## Introduction

The incidence of hepatocellular carcinoma is on the rise worldwide. In addition to hepatitis B and C viruses, alcohol represents a major etiological factor in hepatocarcinogenesis, as shown in numerous epidemiological studies. Alcoholic liver disease is one of the most prevalent liver diseases worldwide, and a major cause of morbidity and mortality.^(1)^ Although important progress has been made in understanding the pathogenesis of alcohol liver disease, the mechanisms involved in its development are not yet fully understood.^(2,3)^ The pathogenesis of alcohol-induced liver disease involves the adverse effects of ethanol metabolites and oxidative stress,^(4,5)^ which is a key step in the pathogenesis of ethanol-associated liver injury.^(4,6)^ Ethanol metabolism leads to the generation of free radicals that can damage cell structure and function. The generation of reactive oxygen species (ROS) increases lipid peroxidation and induces liver dysfunction in rats that have been subjected to acute ethanol exposure.^(6)^ Chronic ethanol consumption increases ROS in the liver and plasma, as demonstrated in animal and clinical studies. Oxidative stress in tissues refers to the generation of ROS and/or a decrease in the level of endogenous antioxidants. Therefore, effective strategies to enhance intracellular antioxidant defences in tissues may help to protect the liver from injury. Antioxidants are essential for preventing cellular damage caused by free radicals. Vitamins E and C are naturally occurring antioxidants that protect against oxidative damage caused by free radicals. Some of these defence systems are impaired after chronic ethanol consumption. Ethanol or its metabolites can alter the redox balance in the liver to a more oxidized state, impairing the antioxidant cell defences. End-stage liver failure is an indication for liver transplantation. It is well known that the liver has a potent regenerative ability.^(7)^ Following partial hepatectomy (PH), the metabolic demands on the liver during regeneration are immense. Liver transplantation for liver disease has satisfactory results. During liver transplantation, reactive oxidative stress is induced by tissue invasion and rapid hemodynamic changes. After liver injury, the liver exhibits active regeneration to repair the injury.^(8)^ Oxidative stress has been considered as a conjoint pathological mechanism, and is believed to contribute to the initiation and progression of liver injury. In this study, we confirmed the effects of two antioxidant vitamins on liver regeneration and the resulting liver function after alcohol administration.

## Materials and Methods

### Animals and Chemicals

A total of 30 adult (7-week-old, weighing approximately 200–210 g) male Sprague-Dawley rats were used. Rats were kept in cages at 22°C with a 12-h light/dark cycle and allowed to consume standard rat pellet chow (Oriental Yeast Co., Ltd., Osaka, Japan) and water ad libitum for 2 weeks. The rats were randomly divided into two groups: alcohol administered group and alcohol non-administered group. The alcohol administered group received 50 ml/L alcohol with the intake being registered daily. Alcohol consumption was calculated in terms of an average of 2.0 g/kg per day. After a certain body weight level (500 ± 10 g) was achieved, two-thirds PH was performed according to the technique by Higgins and Anderson.^(9)^ PH was carried out under light diethyl ether and consisted of the removal of the median and left lateral lobes of the liver.

After surgery, rats were housed individually and pair-fed with rat pellet chow. They were grouped (*n* = 6) as follows: (1) control rats (PH + C) received water ad libitum; (2) PH rats received alcohol solution (PH + A) in their drink containers. PH + A rats were further divided into the following treatment groups: (a) Administration of intraperitoneal dose of vitamin C (PH + A + Vit.C), 250 mg/kg per day; (b) Administration of intraperitoneal dose of vitamin E (PH + A + Vit.E), 250 mg/kg per day; and (c) Administration of intraperitoneal dose of vitamin C and E (PH + A + Vit.C and E), 250 mg + 250 mg/kg per day. All treatments were given daily for five weeks. The weight of each rat was recorded daily.^(10)^

### Biochemical assays

On the first and fifth weeks of treatment, rats were killed using diethyl ether. For biochemical analyses, liver tissue samples were washed with saline and kept frozen at –40°C until the day of the experiment. Hepatic regeneration was determined by the calculation of liver weight regeneration and total DNA concentration.^(11)^ Each post-PH liver remnant was weighed at the time of death. For each rat, the weight of the remnant liver was normalized to the weight of the rat’s entire liver at the time of PH and expressed as the percentage of the rat’s final body weight according to the following formula: (weight of remnant liver/final body weight) × 100.^(12)^ Data from all PH in each treatment group were used to calculate the mean ± SD at the end of the experiment. DNA concentrations were determined fluorometrically according to the methods of Schmidt-Thannhauser-Schneider.^(13)^ Furthermore, RNA concentrations were determined fluorometrically according to the methods of Kamali and Manhouri.^(14)^

Serum was kept frozen at −40°C until use. Alanine aminotransferase (ALT) and aspartate aminotransferase (AST) activities, as indicators of the extent of liver injury, were measured. Serum albumin and total bilirubin concentrations were measured to assess liver dysfunction.

Serum was kept frozen at −80°C until use. The difference in optical density at 532 nm was measured in terms of the serum MDA (malondialdehyde) content, an index of lipid peroxidation.^(15)^

### Statistical analysis

All data are expressed as mean ± SD. Statistical analysis was performed using the Student’s *t* test and Tukey’s multiple range test.^(16)^ Differences between groups were considered to be significant at *p*<0.05.

## Results

**Fig. 1:**
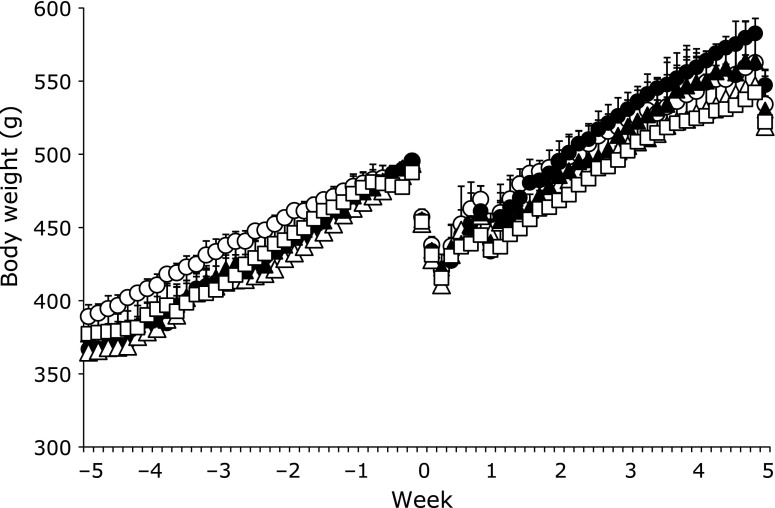
The effect of alcohol and vitamin administration on body weight before and after partial hepatectomy. ◯, Partial hepatectomy + Control (PH + C); ●, Partial hepatectomy + Alcohol (PH + A); △, Partial hepatectomy + Alcohol + Vitamin C (PH + A + Vit.C); ■, Partial hepatectomy + Alcohol + Vitamin E (PH + A + Vit.E); □, Partial hepatectomy + Alcohol + Vitamin C and E (PH + A + Vit.C and E) groups. Data represent means ± SD. *p<0.05.

The effect of the combination of alcohol and vitamins on the body weight of the rats is shown in Fig. 1. Rats in the alcohol-administered groups had a higher body weight than the PH-only group.

**Fig. 2:**
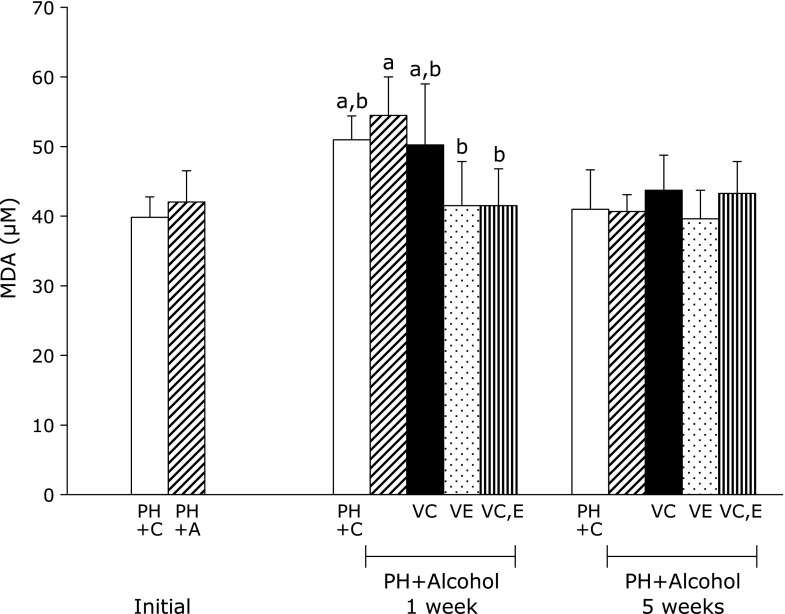
Malondialdehyde (MDA) levels in rat serum 1 week and 5 weeks after partial hepatectomy and daily ingestion of alcohol and administration of vitamins C and/or E. Partial hepatectomy + Control (PH + C), Partial hepatectomy + Alcohol (PH + A), Partial hepatectomy + Alcohol + Vitamin C (PH + A + Vit.C; VC), Partial hepatectomy + Alcohol + Vitamin E (PH + A + Vit.E; VE), Partial hepatectomy + Alcohol + Vitamin C and E (PH + A + Vit.C and E; VC,E) groups. All values are expressed as means ± SD. Different superscript letters indicate significant differences for each group (*p*<0.05).

To evaluate the damage produced by ROS, the serum MDA concentration was determined in rats treated with alcohol and vitamins (Fig. 2). A higher serum MDA concentration was observed in the PH + A group than in the control PH group. This difference became more evident after administration of alcohol for 1 week. With vitamin C administration, as in the PH + A + Vit.C group, the serum MDA level was similar to that in the PH + C group at 1 week.

The PH + A + Vit.E and the PH + A + Vit.C and E groups had significantly lower serum concentrations of MDA, being comparable to that in the PH + A group 1 week after PH. There were no significant differences in serum MDA observed among groups 5 weeks after PH.

**Fig. 3:**
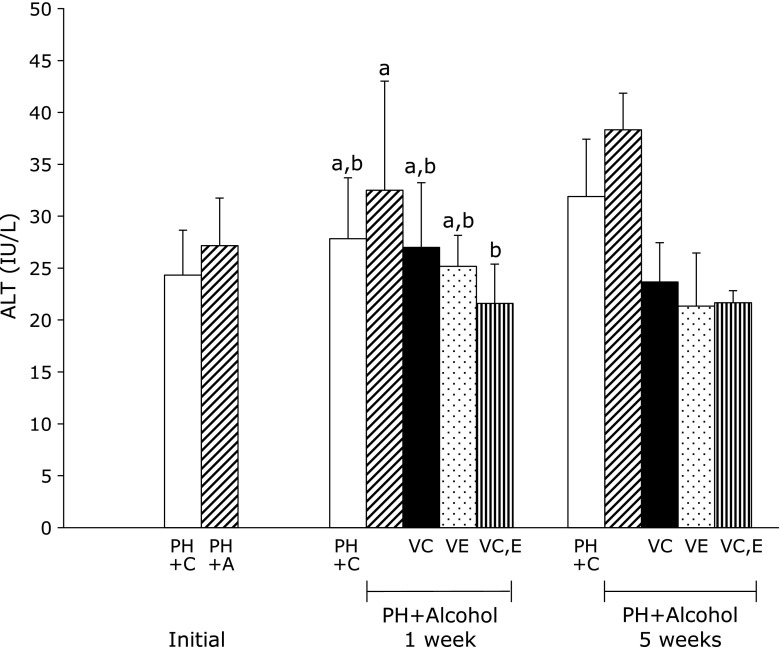
Serum alanine aminotransferase (ALT) levels in rats 1 week and 5 weeks after partial hepatectomy and daily ingestion of alcohol and administration of vitamins C and/or E. Partial hepatectomy + Control (PH + C), Partial hepatectomy + Alcohol (PH + A), Partial hepatectomy + Alcohol + Vitamin C (PH + A + Vit.C; VC), Partial hepatectomy + Alcohol + Vitamin E (PH + A + Vit.E; VE), Partial hepatectomy + Alcohol + Vitamin C and E (PH + A + Vit.C and E; VC,E) groups. All values are expressed as means ± SD. Different superscript letters indicate significant differences for each group (*p*<0.05).

**Fig. 4:**
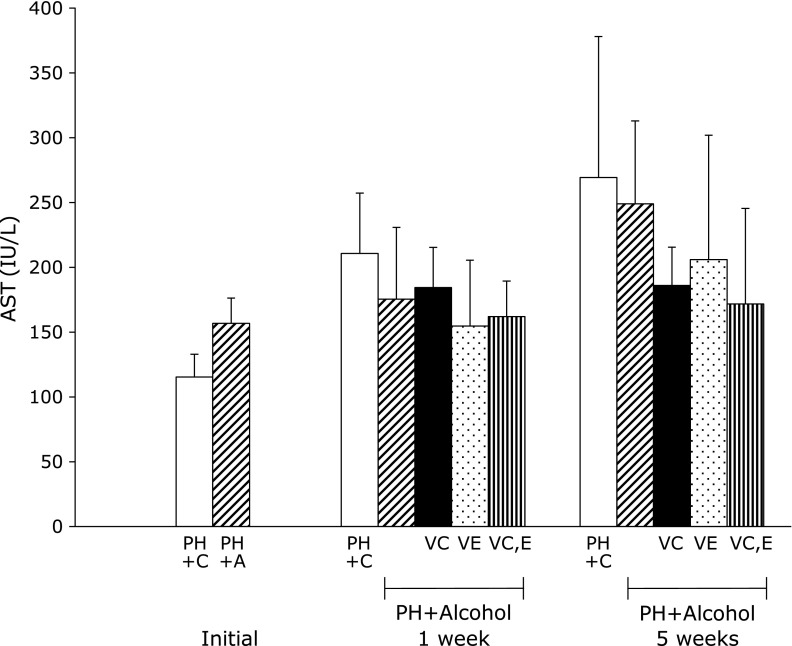
Serum aspartate aminotransferase (AST) levels in rats 1 week and 5 weeks after partial hepatectomy and daily ingestion of alcohol and administration of vitamins C and/or E. Partial hepatectomy + Control (PH + C), Partial hepatectomy + Alcohol (PH + A), Partial hepatectomy + Alcohol + Vitamin C (PH + A + Vit.C; VC), Partial hepatectomy + Alcohol + Vitamin E (PH + A + Vit.E; VE), Partial hepatectomy + Alcohol + Vitamin C and E (PH + A + Vit.C and E; VC,E) groups. All values are expressed as means ± SD.

Concerning the protective effects of vitamins C and E on liver function, the PH + A group had slightly elevated serum concentration of ALT compared with the PH + C group (Fig. 3). The three groups with alcohol and vitamin administration had lower serum concentrations of ALT (Fig. 3) and AST (Fig. 4) than the PH + A group. The PH rats treated with combinations of alcohol and vitamins C and E had significantly lower levels of serum concentrations of ALT, being comparable to the control PH + C group. A similar effect was observed in rats treated with alcohol and vitamin C (PH + A + Vit.C), suggesting the low potential of this vitamin in promoting hepatic regeneration.

Table 1 shows the results of indicators associated with hepatic regeneration: liver/body ratio, mgDNA/g liver tissue and mgRNA/g liver tissue. Lower levels of hepatic DNA were observed in the PH + A group than in the PH + C group 1 week after PH (Table 1A). Interestingly, the three groups with combinations of alcohol and vitamin administration showed higher levels of hepatic DNA than the PH + A group. However, hepatic RNA levels were reduced in the alcohol-treated groups 5 weeks after PH (Table 1B). When vitamin C and/or E was administered, hepatic RNA was similar to that of the PH + C group. In the PH + A group there was no difference in liver/body ratio compared with the PH + C group at 5 weeks after treatment (Table 1B). However, the PH + A + Vit.E group and the PH + A + Vit.C and E group showed higher liver/body ratios than the PH + A group. Treatment with vitamin C and vitamin E may have promoted liver regeneration.

**Fig. 5:**
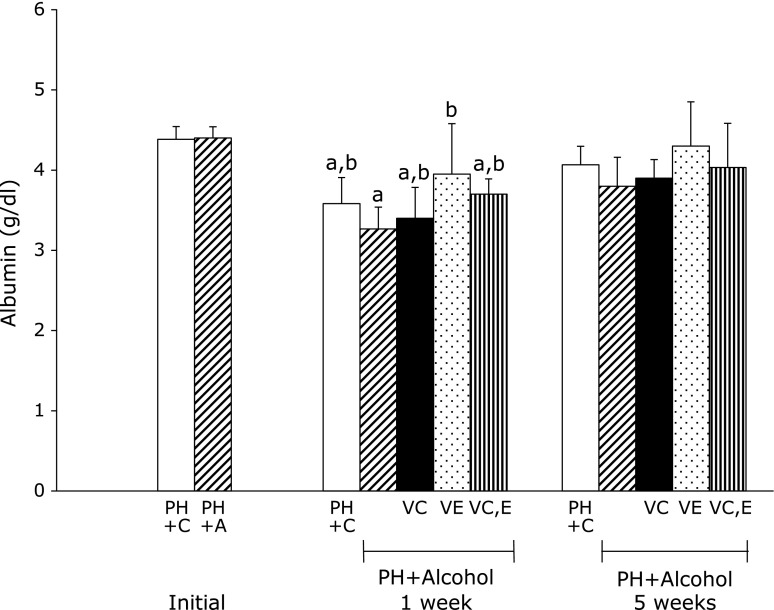
Albumin levels in serum from rats 1 week and 5 weeks after partial hepatectomy and daily ingestion of alcohol and administration of vitamins C and/or E. Partial hepatectomy + Control (PH + C), Partial hepatectomy + Alcohol (PH + A), Partial hepatectomy + Alcohol + Vitamin C (PH + A + Vit.C; VC), Partial hepatectomy + Alcohol + Vitamin E (PH + A + Vit.E; VE), Partial hepatectomy + Alcohol + Vitamin C and E (PH + A + Vit.C and E; VC,E) groups. All values are expressed as means ± SD. Different superscript letters indicate significant differences for each group (*p*<0.05).

**Fig. 6:**
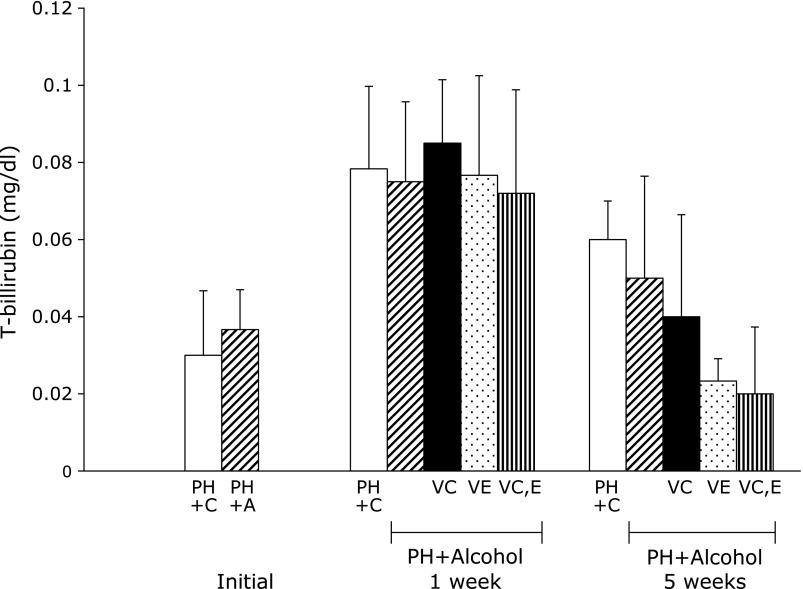
Total bilirubin (T-bilirubin) levels in serum from rats 1 week and 5 weeks after partial hepatectomy and daily ingestion of alcohol and administration of vitamins C and/or E. Partial hepatectomy + Control (PH + C), Partial hepatectomy + Alcohol (PH + A), Partial hepatectomy + Alcohol + Vitamin C (PH + A + Vit.C; VC), Partial hepatectomy + Alcohol + Vitamin E (PH + A + Vit.E; VE), Partial hepatectomy + Alcohol + Vitamin C and E (PH + A + Vit.C and E; VC,E) groups. All values are expressed as means ± SD.

Figure 5 and 6 show the results of liver metabolic integrity. In the PH + A group, low serum levels of albumin were evident compared with PH + C group 1 week after PH. Serum albumin in PH + A + Vit.E group was significantly higher than that in the PH + A group. Serum total bilirubin levels 1 week after treatment were similar in PH + C and PH + A groups. Administration of vitamins C and E led to lower serum levels of total bilirubin compared with the PH + A group 5 weeks after PH.

## Discussion

The first successful living-donor liver transplantation (LDLT) was performed in 1989 on a child in Japan.^(17)^ Since then, the LDLT survival rate has risen annually, with a 5-year patient survival rate of 70% in 2013.^(18)^ These satisfactory results reflect the significant advancement in liver transplantation techniques. However, recent studies have demonstrated that a residual effect on liver function is a barrier to satisfactory results;^(19–26)^ namely, at the time of PH, ROS and lipid peroxide produced by acute hemodynamic changes and tissue invasion affect the residual liver function. Ethanol metabolism generates ROS, which impaires the antioxidant defense system, leading to increased lipid peroxidation.^(27,28)^ This increased peroxidation is due to the increased oxidative stress in hepatic tissues induced by ethanol and its oxidation.^(29,30)^ Increased oxidative stress is known to lead to hepatocyte damage.^(24,29–31)^ Consequently, early removal of ROS and lipid peroxidation may improve the prognosis after PH and transplantation.^(25,31)^ Also, postoperative general complications and postoperative fatigue syndrome (POFS) are adverse symptoms that are often reported to have a significant impact on the patient’s quality of life.^(32)^ The symptoms of POFS may be prolonged, increasing the cost of medical services and often placing a heavy burden on the patient, their family and society. Improvement measures are thus also necessary to expedite the return to daily life. The aim of the present study was to determine whether a combination of vitamins C and E had protective effects on ethanol-induced changes in lipid peroxidation^(31,33–37)^ and MDA levels in rat liver tissues and serum parameters following ethanol-induced hepatic dysfunction.^(19,21–24)^

Kishino reported that PH causes biochemical changes in the intrahepatic fatty acid composition.^(38)^ After hepatectomy, membrane-bound phospholipase A2 is activated as an inducer of hepatic regeneration.^(39,40)^ It degrades the phospholipid membrane, leading to the release of large quantities of polyunsaturated fatty acids (PUFAs).^(39,40)^ PUFAs are particularly susceptible to oxidation by free radicals and other highly-reactive species.^(41)^ ROS, such as the hydroxyl radical (^•^OH), lead to the formation of lipid peroxyl radicals (LOO^•^),^(41)^ which react with a second PUFA, forming a lipid hydroperoxide (LOOH) and a second LOO^•^, resulting in further lipid oxidation.^(42,43)^ Alternatively, LOO^•^ attack an intramolecular double bond and form a cyclic endoperoxide, which decomposes to MDA.^(41)^ MDA is one of many low molecular weight end-products of lipid hydroperoxide decomposition and is the most often utilized as an index of lipid peroxidation.^(44)^ An increase in the serum MDA concentration was observed in the PH + A group compared with the PH + C group 1 week after PH. This result suggests that the effect of PH, as well as continuous alcohol administration, increases lipid peroxidation. Alcohol-induced oxidative damage also causes alterations in phospholipids and consequent changes in the structural characteristics and dynamics of the lipid bilayer, with functional impairment in membrane fluidity, ion permeability, membranous enzyme activity, and cell signalling.^(45)^ The continuous infusion of alcohol may increase the induction of cytochrome P-450 2E1 (CYP2E1) and generate superoxide (O_2_^•^–).^(5,28,46,47)^ Superoxide dismutase (SOD) promotes the conversion of superoxide (O_2_^•−^) to hydrogen peroxide (H_2_O_2_). Furthermore, highly-reactive hydroxyl radicals (HO^•^) are produced in the presence of iron (Fe).^(28)^ These hydroxyl radicals (HO^•^) induce oxidative stress, which results in lipid peroxidation in biological membranes.^(24,28)^ Bailey and Cunningham have proposed that the excess of reducing equivalents generated when ethanol is oxidized by liver alcohol dehydrogenase produces a more reduced electron transfer chain, which will facilitate the transfer of electrons to molecular oxygen to produce superoxide.^(48–50)^ ROS generation will be further elevated after chronic ethanol consumption because of the decreased activity of the respiratory chain, resulting in accumulation of reduced respiratory carriers in complexes I and III.^(50,51)^ Increased ROS production by ethanol metabolism has been reported to be associated with a slight decrease in hepatocyte survival and an increase in mitochondrial protein carbonyl levels reflecting oxidized protein accumulation.^(50,52)^ Thus, the mitochondria contribute to the increase in oxidant levels in hepatocytes exposed acutely or chronically to ethanol.^(50)^ Damage to the mitochondria plays a critical role in the CYP2E1 plus iron-dependent toxicity. CYP2E1 generates ROS, such as O_2_^•−^ and H_2_O_2,_ during its catalytic cycle.^(50)^ Iron promotes oxidative stress by catalyzing the conversion of less reactive oxidants, such as superoxide or H_2_O_2,_ to more powerful oxidants such as hydroxyl radical or perferryl-type oxidants.^(53)^ Increased lipid peroxide initiates oxidative DNA damage and parenchymal hepatocyte injury.^(24,28,54–56)^ It has also been observed that an increase in lipid peroxidation impairs liver regeneration.^(21–25)^ Similar results were observed in this study.

Bradford reported that activation of CYP2E1 is important for ethanol-induced DNA damage in the liver and ethanol-related hepatocellular carcinogenesis.^(57)^ If an injury involves other organelles, such as mitochondria, soluble enzymes, such as AST and ALT, will also be released into the blood, indicating that PH causes cellular damage,^(11,58)^ and reduces residual liver function.^(59)^ ALT is predominantly found in the liver, whereas AST is found in many organs including the liver, cardiac muscle, skeletal muscle and erythrocytes. The enzymatic activities of these two enzymes are indicators of parenchymal hepatocyte proliferation damage.^(59)^ Increased alcohol metabolism contributes to liver damage. Chronic alcohol consumption leads to the leakage of cell contents into the plasma. Alcohol consumption reacts with free amino, sulfhydryl and other functional groups to alter the membrane composition, resulting in oxidative modification of proteins and consequent instability of the membrane.^(45,60)^ The high content of iron and PUFAs in erythrocytes makes them highly susceptible to alcohol induced ROS and oxidative damage.^(45)^ Free radicals such as ROS can directly damage erythrocyte membranes by lipid peroxidation of membrane PUFAs.^(45)^ Chronic alcohol consumption causes changes in erythrocyte structure, and as decomposition of erythrocytes increases due to blood flow disturbance, liver dysfunction results in anemia and jaundice.^(45)^ The PH + A group tended to have elevated serum concentrations of pre-operative ALT and AST compared with the PH + C group. After 1 week, the serum concentration of ALT was significantly different between PH + A and PH + A + Vit.C and E groups. After 5 weeks, the PH + C and PH + A groups had elevated serum concentrations of ALT compared with the administration of vitamins A, E and C in the ethanol groups, whereas rats in the PH + A + Vit.C, PH + A + Vit.E and PH + A + Vit.C and E groups maintained similar ALT levels. In addition to hepatectomy and alcohol consumption, enzymes may be released due to destruction of hepatocytes or erythrocytes and lead to hemolysis. Administration of vitamins C and E led to lower serum levels of total bilirubin compared with the PH + A group 5 weeks after PH. In conclusion, Vitamins C and E administration attenuates hepatocytes and erythrocyte destruction and contributes to the recovery of postoperative liver function. We believe that vitamins C and E may play a role in the suppression of hepatic indicators of parenchymal hepatocyte proliferation in an indirect manner.^(61,62)^ We speculate that vitamins C and E inhibit lipid peroxidation after PH, and suppress the increase in serum AST and ALT activation. Improving residual liver function can enhance hepatic regeneration. After 5 weeks, a marked difference in AST levels was evident compared with 1 week after PH.

In all groups, the total serum bilirubin levels 1 week after PH were higher than the initial levels immediately after PH. Recent studies have shown that bilirubin is a potent antioxidant.^(63,64)^ Thus, the total bilirubin levels were elevated by removal of ROS during the liver regeneration process, transiently contributing to liver regeneration. After 5 weeks, low total serum bilirubin levels were found (from highest to lowest) in PH + A, PH + A + Vit.C, PH + A + Vit.E, and PH + A + Vit.C and E groups. The results suggest that postoperative vitamin C and E administration influences lipoperoxidative reactions during liver regeneration. After 1 week, hepatic RNA was slightly higher in vitamin-administered groups than in PH + C and PH + A groups. However, there were no differences in hepatic RNA among the groups after 5 weeks. Therefore, these vitamins may increase protein synthesis in the liver.^(65)^ After liver regeneration by increased synthesis of cellular proteins, albumin synthesis was elevated.^(66)^ Thus, the postoperative reduction in serum albumin levels caused by PH recovered, and the levels were elevated in all groups after 5 weeks.

It appears that vitamins C and E administration promotes liver regeneration. Vitamin E has a suppressive effect on the increase in lipid peroxidation by inhibiting lipoperoxidative chain reactions. Recent studies have demonstrated that vitamins C and E have a marked synergistic antioxidant action.^(67,68)^ Similar results were observed in this study. In this experiment, vitamin C and vitamin E were administered at doses of 250 mg/kg body weight per day. It is possible that the action of these vitamins is dose-dependent. Based on our findings, it is likely that administration of vitamins C and E markedly promotes liver regeneration and reduces parenchymal hepatocyte damage after PH and continuous alcohol administration. Additional studies are required, such as assessing the effects of oral administration of vitamins C and E, as well as the amount of alcohol administered. We also need to examine the residual liver function of the rat over a longer period of time. Based on the ratio of liver mass relative to body weight, a possible influence of vitamins C and E on the promotion of liver regeneration cannot be excluded.^(29)^ Our results suggest that vitamins C and E affect liver regeneration by enhancing the functional role of hepatocytes. Lu^(32)^ reported a metabolomics analysis in POFS rats induced by PH. The citric acid cycle, branched-chain amino acids metabolism, fatty acid transport and metabolism, phospholipid metabolism, tryptophan metabolism, phenylalanine metabolism and purine metabolism became abnormal as metabolic pathways and potential biomarkers associated with POFS. Among these results, it is necessary to supplement the branched chain amino acids reduced by fatigue-induced glycogen depletion. This supplementation is useful for relieving fatigue and promoting recovery after surgery. Whether this also contributes to the protection of residual liver function should be studied. There is a need to clarify basic information on the role of oxidative stress in the onset of disease and ROS-related cytotoxicity and to continue to consider reasonable antioxidant therapies in the future.

## Figures and Tables

**Table 1:** Evaluation parameters involved in liver regeneration in each experimental group after (A) 1 week and (B) 5 weeks of treatment with partial hepatectomy (PH), alcohol (A) and vitamins

**A**					

Group	Final body weight (g)	Final liver weight (g)	Liver/body ratio (%)	mgDNA/g liver tissue	mgRNA/g liver tissue

PH + C (*n* = 3)	435.9 ± 1.9	15.1 ± 0.6	3.5 ± 0.2	3.7 ± 0.4	10.5 ± 1.8
PH + A (*n* = 3)	421.8 ± 5.2	14.2 ± 1.5	3.4 ± 0.3	2.8 ± 0.4	10.9 ± 2.1
PH + A + Vit.C (*n* = 3)	449.1 ± 5.8	13.7 ± 1.2	3.1 ± 0.3	3.9 ± 0.5	13.4 ± 2.1
PH + A + Vit.E (*n* = 3)	440.7 ± 2.0	15.5 ± 0.8	3.5 ± 0.2	4.4 ± 0.8	14.2 ± 3.8
PH + A + Vit.C and E (*n* = 3)	416.3 ± 3.4	17.1 ± 0.6	4.1 ± 0.1	4.1 ± 0.1	13.7 ± 3.8

**B**					

Group	Final body weight (g)	Final liver weight (g)	Liver/body ratio (%)	mgDNA/g liver tissue	mgRNA/g liver tissue

PH + C (*n* = 3)	528.0 ± 19.6	20.5 ± 1.7	3.9 ± 0.2	2.9 ± 0.1	10.3 ± .3.7
PH + A (*n* = 3)	547.2 ± 6.2	19.5 ± 2.7	3.6 ± 0.5	3.0 ± 0.7	9.4 ± 1.8
PH + A + Vit.C (*n* = 3)	498.9 ± 24.8	18.7 ± 2.7	3.7 ± 0.4	3.7 ± 0.3	10.6 ± 2.4
PH + A + Vit.E (*n* = 3)	530.3 ± 9.6	23.6 ± 0.2	4.4 ± 0.1	3.9 ± 0.2	11.5 ± 1.5
PH + A + Vit.C and E (*n* = 3)	496.2 ± 35.9	22.5 ± 2.7	4.5 ± 0.2	4.1 ± 0.7	11.0 ± 2.0

Values are presented as means ± SD for each experimental group (*n* = 3). Partial hepatectomy + Control (PH + C), Partial hepatectomy + Alcohol (PH + A), Partial hepatectomy + Alcohol + Vitamin C (PH + A + Vit.C), Partial hepatectomy + Alcohol + Vitamin E (PH + A + Vit.E), Partial hepatectomy + Alcohol + Vitamin C and E (PH + A + Vit.C and E) groups.
